# The Medial Thalamus Plays an Important Role in the Cognitive and Emotional Modulation of Orofacial Pain: A Functional Magnetic Resonance Imaging-Based Study

**DOI:** 10.3389/fneur.2020.589125

**Published:** 2021-01-21

**Authors:** Yu Jin, Hong Yang, Feifei Zhang, Jue Wang, He Liu, Xin Yang, Hu Long, Fei Li, Qiyong Gong, Wenli Lai

**Affiliations:** ^1^State Key Laboratory of Oral Disease, Department of Orthodontics, West China School of Stomatology, Sichuan University, Chengdu, China; ^2^Department of Radiology, Huaxi MR Research Center (HMRRC), West China Hospital of Sichuan University, Chengdu, China; ^3^West China School of Stomatology, Sichuan University, Chengdu, China; ^4^Department of Orthodontics, China-Japan Friendship Hospital, Beijing, China; ^5^Department of Stomatology, Shanghai Jiao Tong University School of Medicine Xinhua Hospital, Shanghai, China; ^6^Psychoradiology Research Unit of the Chinese Academy of Medical Sciences (2018RU011), West China Hospital of Sichuan University, Chengdu, China

**Keywords:** orofacial pain, functional connectivity, perception, thalamic subregions, functional magnetic resonance imaging-fMRI, fractional amplitude of low-frequency fluctuations-fALFF

## Abstract

The thalamus plays a critical role in the perception of orofacial pain. We investigated the neural mechanisms of orofacial pain by exploring the intrinsic functional alterations of the thalamus and assessing the changes in functional connectivity (FC) between the thalamic subregions with significant functional alterations and other brain regions in orofacial pain using the seed-based FC approach. There were 49 participants in the orofacial pain group and 49 controls. Orofacial pain was caused by orthodontic separators. The resting-state functional magnetic resonance imaging data of the two groups were analyzed to obtain the fractional amplitude of low-frequency fluctuations (fALFF) of the thalamus; the thalamic subregions with significant fALFF abnormalities were used as seeds for FC analysis. Student's *t*-tests were used for comparisons. Pearson's correlation analysis was performed using SPM software. Forty-four participants with orofacial pain (mean age, 21.0 ± 0.9 years; 24 women) and 49 age- and sex-matched controls (mean age, 21.0 ± 2.6 years; 27 women) were finally included. Compared with the control group, the orofacial pain group demonstrated the following: (1) increased function in the dorsal area of the thalamus and decreased function in the medial thalamus; (2) decreased FC between the medial thalamus and 12 brain regions (*p* < 0.05, family-wise error corrected, voxel > 100); and (3) potential positive and negative correlations between the medial thalamus-seeded FC and visual analog scale score changes (*p* < 0.05, AlphaSim corrected). The findings show that the medial and dorsal thalami play important roles in orofacial pain perception, and that the medial thalamus likely plays an important role in the cognitive and emotional modulation of orofacial pain.

## Introduction

Orofacial pain, an ache localized in the oral and facial regions (1), is a symptom of a variety of diseases, such as pulpitis, periodontitis, and temporomandibular joint disorder (TMD) (2). It is a common condition; studies in Brazil (3) and Hong Kong (4) found that over 40% of the population had experienced orofacial pain. Because of the complexity of the affected regions, orofacial pain can have a deleterious effect on the patients' daily functions (including eating, drinking, and speaking) and can seriously affect various social functions and the quality of life (5, 6).

It is well-known that the trigeminal nerve contains most sensory nerve fibers innervating orofacial tissues and is important for orofacial pain perception. Once stimulated, the peripheral nerves transmit nociceptive information to the central nervous system. The trigeminal primary afferent projects the information to second-order neurons located in the trigeminal brainstem sensory nuclear complex through the trigeminal ganglion. The trigeminal nucleus sends the information in the ventroposterior nucleus of the thalamus, and the thalamus relays it to the cortex (7).

In the abovementioned pathways, the thalamus is a key node. It serves as a global hub, connected with the entire cortex, relaying sensory information to the cortex, and mediating the transmission of cortico-cortical information (8, 9). Moreover, it is important in both nociceptive transmission and pain modulation (10, 11). Furthermore, the thalamus can be divided into several subregions anatomically and functionally as a heterogeneous structure (12, 13). Although it is well documented that it plays a critical role in orofacial pain perception, there is limited imaging evidence of how the thalamus and its subregions contribute to orofacial pain perception.

Functional magnetic resonance imaging (fMRI) is used to measure neural activity by detecting spontaneous blood oxygen level–dependent fluctuations (14). In recent years, fMRI has been used to study the neural mechanism of orofacial pain (including TMD, trigeminal neuralgia, and orthodontic pain), and the authors identified some significant abnormalities in brain functions and functional connectivity (FC) (15–17). However, no fMRI studies have focused on the functional and FC changes of the thalamus in orofacial pain.

Orthodontic elastic separators are usually used to put spaces for installation of orthodontic bands before orthodontic treatment and have also been used to develop pain research models in some previous studies (18–21). Therefore, we created a model of orofacial pain by placing orthodontic elastic separators to the teeth, and explored the intrinsic functional alterations of the thalamus and used the seed-based FC approach to analyze resting-state fMRI (rfMRI) data to assess the changes in FC between the thalamic subregions that showed significant functional alterations and other brain regions in orofacial pain.

## Materials and Methods

### Subjects

This prospective study was approved by the Ethics Committee of the local institution, and all participants provided written informed consent before enrollment. All procedures were in line with the tenets of the Declaration of Helsinki.

All participants were recruited after meeting the following inclusion criteria: were right handed adults; had bachelor degree or above; no serious malocclusion, temporomandibular joint disease, or other diseases causing orofacial pain; no history of drug or alcohol abuse; no history of neurological disorders; and no contraindications for MRI, including cardiac pacemakers and other metallic implants. The orofacial pain group included 49 subjects, in whom orofacial pain was induced by placement of orthodontic elastic separators. The control group consisted of 49 age- and sex-matched control subjects.

### Study Design

Orofacial pain was caused by placement of orthodontic elastic separators to the mesial sides of the left lower first molar in the orofacial pain group (2). The elastic separator (Morelli, 4.0 mm, Ø 5/32”, reference # 60.04.200) was stretched with two pieces of dental floss and seesawed between two teeth until there was a click. This operation was performed by a trained orthodontist. rfMRI was performed after 24 h because orofacial pain peaks at 24 h after placement of the separator (22). The controls underwent rfMRI scan without separators. A 100-mm visual analog scale (VAS, ranging from 0 to 100) and the Symptom Checklist-90-Revision (SCL-90-R) were used for degree of discomfort measurement and psychological evaluation, respectively, both before the elastic separator placement and before the scan. Patients experiencing greater discomfort tend to have higher VAS scores. The elastic separators were removed after the scan.

### MRI Data Acquisition

All participants underwent rfMRI. Imaging was performed with a Siemens 3.0-T MRI system (Trio; Siemens, Erlangen, Germany). During data acquisition, all participants were instructed to rest with their eyes closed, not asleep, and not to move their heads or think of anything in particular. Each participant's head was fixed with foam pads to minimize head motion. The fMRI scan parameters were as follows: repetition time (ms)/echo time (ms), 2,000/30; flip angle, 90°; field of view, 240 × 240 mm^2^; matrix size, 64 × 64; voxel size, 3.75 × 3.75 × 5 mm^3^; and section thickness, 5 mm; 205 volumes were obtained.

### Image Processing and Data Analysis

MRI data analysis was performed using software (SPM8, Statistical Parametric Mapping, http://www.fil.ion.ucl.ac.uk/spm/software/spm8). For image preprocessing, the initial 10 volumes were discarded to obtain a steady state of the resting condition. Thus, we analyzed the remaining 195 volumes. Head translation movement of the participants who were finally included was <2.5 mm and the rotation was <2.5° (23). Nuisance signal, including white matter and cerebrospinal fluid signal, and 24-parameter motion correction were regressed out. The linear regression model with motion “spike” as a separate regressor was used for temporal scrubbing. We defined the motion “spike” as the time point with a high framewise displacement (>1), and there was no significant difference in mean FD between orofacial pain group and control group (0. 167 mm ± 0.098 vs. 0.171 mm ± 0.102, *t* = −0.180, *p* = 0.857). Next, the resulting functional images were registered to each individual's 3D T1 structural images. Images were normalized to the standard space of the Montreal Neurological Institute using affine transformation and non-linear deformations (24), and each voxel was resampled to 3 × 3 × 3 mm^3^.

### Fractional Amplitude of Low-Frequency Fluctuations Analyses

The REST (http://www.restfmri.net/forum, version 1.8) software was used to calculate the fractional amplitude of low-frequency fluctuations (fALFF) of the thalamus. We transformed the time series into a frequency domain power spectrum after preprocessing. Subsequently, we performed the root-mean-square calculation on the power spectrum in the range of 0.01–0.1 Hz, and the ALFF value was obtained. The fALFF value was computed as a ratio of the sum of the amplitudes of the entire low-frequency band (0.01–0.1 Hz) to that of the whole detected frequency range. Finally, the fALFF value of each voxel was divided by the whole brain mean fALFF value in order to normalize all subject data for standardization. Then we extracted the thalamus from the Automated Anatomic Labeling (AAL90) template as a mask and only focused on the functional changes in the area where the thalamus was located for our comparisons.

### Seed-Based FC Analyses

As we report below, significant fALFF abnormalities in the orofacial pain group were demonstrated in two thalamic subregions, the medial thalamus and the dorsal area of the thalamus, and these two subregions were chosen as seeds for seed-based FC analysis by using REST.

We averaged the rfMRI time series of voxels in each seed to extract the reference time series for each seed. A Pearson correlation analysis was performed to analyze the association between each seed region with that of other voxels across the rest of the brain to generate FC maps (25). Fisher's *r*-to-*z* transformation was used to convert the distribution of the correlation coefficients (*r*) into a normal distribution. Automated Anatomical Labeling atlas (https://www.oxcns.org/aal.html) was used to determine the anatomical boundaries of areas that showed significant changes in FC to the medial thalamus or the dorsal area of the thalamus.

### Statistical Analyses

Statistical analyses were performed using SPSS software (version 20.0; SPSS, Chicago, IL). The two-sample *t*-tests were performed to compare the differences of the variables including age, FD, VAS, and SCL-90-scores before the elastic separator placement between the subjects in orofacial pain group and controls. And we used chi-square test to compare the differences of sex ratio between the two groups. In the orofacial pain group, a paired *t*-test was used to compare the VAS and SCL-90-R scores before the elastic separator placement and before the scan. The comparison of fALFF and FC differences between subjects with orofacial pain and control subjects was performed with a two-sample *t*-test using SPM8, with age, sex and FD as covariates, and a significance threshold of *p* = 0.05. False discovery rate (FDR) at voxel level correction was used to conduct the multiple comparisons in fALFF analysis, while family-wise error (FWE) correction at voxel level was used for multiple comparisons in FC analysis. In the orofacial pain group, we performed a multiregression analysis using SPM software to determine whether the FC was associated with pain intensity changes, with age, sex and FD as covariates, corrected for multiple comparisons with AlphaSim correction. Specifically, the threshold for significant clusters was set as follows: 1,000 iterations, *p* < 0.05 at the cluster level combined with *p* < 0.05 at the voxel level, 4 estimated smooth kernels, at least 154 voxels.

## Results

### Patients' Demographics

Forty-nine individuals with orofacial pain and 49 age- and sex-matched control subjects were enrolled. We excluded five participants with orofacial pain who had excessive head movement of >2.5 mm or 2.5° during imaging. Thus, 44 participants in the orofacial pain group (24 females and 20 males) and 49 controls (27 females and 22 males) were included in the analysis. There were no significant differences in the age distribution (21.0 ± 0.9 vs. 21.0 ± 2.6 years, *t* = −0.11, *p* = 0.991) or sex ratio (χ^2^ = 0.003, *p* = 0.957), VAS score (14.7 ± 17.0 vs. 13.7 ± 16.4, *t* = 0.296, *p* = 0.768) or SCL-90-R score (27.7 ± 11.0 vs. 26.4 ± 11.1, *t* = 0.566, *p* = 0.573) before the elastic separator placement between the orofacial pain and control groups (Table 1).

**Table 1:** Demographic variables in orofacial pain group and control group.

	**Orofacial pain group** ** (*n* = 44)**	**Control group** ** (*n* = 49)**	***t*/χ^**2**^**	***p*-value**
Age (mean ± SD)	21.0 ± 0.9	21.0 ± 2.6	−0.1	0.991
Sex ratio (mean ± SD)	24/20	27/22	0.003	0.957
FD (mean ± SD)	0.167 ± 0.098	0.171 ± 0.102	0.180	0.857
VAS(mean ± SD)	14.7 ± 17.0	13.7 ± 16.4	0.296	0.768
SCL-90-R (mean ± SD)	27.7 ± 11.0	26.4 ± 11.1	0.566	0.573

*FD, framewise displacement; VAS, visual analog scale; SCL-90-R, Symptom Checklist-90-Revision*.

*Values are presented as mean ± standard deviations*.

And in the orofacial pain group, the VAS score before the scan was significantly increased compared with that before the elastic separator placement (6.8 ± 16.7, *t* = −2.7, *p* = 0.01). The mean *S*-scores of the SCL-90-R before the elastic separator placement and before the scan were 27.7 ± 11.0 and 29.3 ± 10.5, respectively, with no statistically significant difference (*t* = −1.3, *p* = 0.206; Table 2).

**Table 2:** Pain intensity and psychological evaluation of the participants with orofacial pain.

	**Pairing difference**	***t*-value**	***p*-value**
VAS (mean ± SD)	6.8 ± 16.7	−2.7	0.010
SCL-90-R (mean ± SD)	1.6 ± 8.2	−1.3	0.206

*VAS, visual analog scale; SD, standard deviation; SCL-90-R, Symptom Checklist-90-Revision*.

*Values are presented as mean ± standard deviations*.

### fALFF Differences and Seed-Based FC

**Figure 1:**
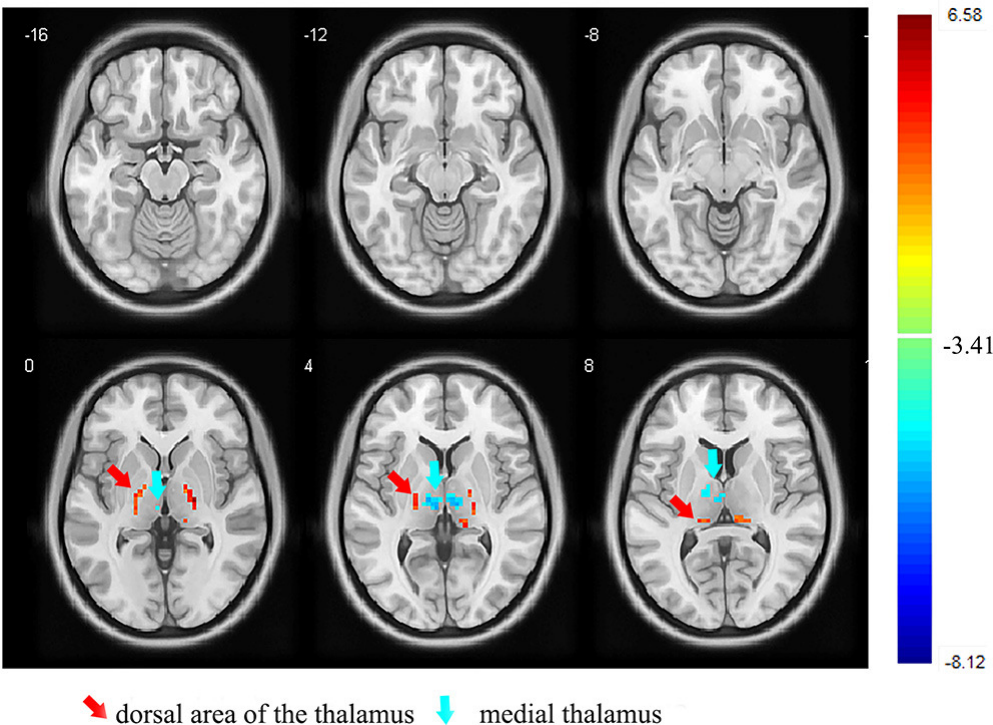
Images showing the results of the fALFF analysis. Compared with the controls, the participants with orofacial pain showed a decreased fALFF (blue) in the medial thalamus and increased fALFF (red) in the dorsal area of the thalamus. fALFF, fractional amplitude of low-frequency fluctuation (*p* < 0.05, FDR corrected). At right, color bars indicate *t-*values.

First, we examined the differences between the two groups in the whole-thalamus fALFF. The results showed that the fALFF of the medial thalamus was significantly reduced, and that of the dorsal area of the thalamus was significantly increased compared with those of the control group (*p* < 0.05, FDR corrected; Figure 1 and Table 3).

**Table 3:** Significant differences in regional fALFF between the orofacial pain and control groups.

**Brain region**	**MNI coordinates**			**Clusters size**	***t-*Value**
	***x***	***y***	***z***		
**Regions of thalamus showing increased fALFF in orofacial pain group relative to control group**					
Right dorsal thalmus	9	−21	12	68	−7.89
Left dorsal thalmus	−12	−27	12	35	−5.43
**Regions of thalamus showing decreased fALFF in orofacial pain group relative to control group**					
Right medial thalmus	9	−21	3	51	6.56
Left medial thalmus	−6	−15	6	34	6.58

*The fALFF of the medial thalamus was significantly reduced, and that of the dorsal area of the thalamus was significantly increased compared with those of the control group (p < 0.05, FDR corrected)*.

**Figure 2:**
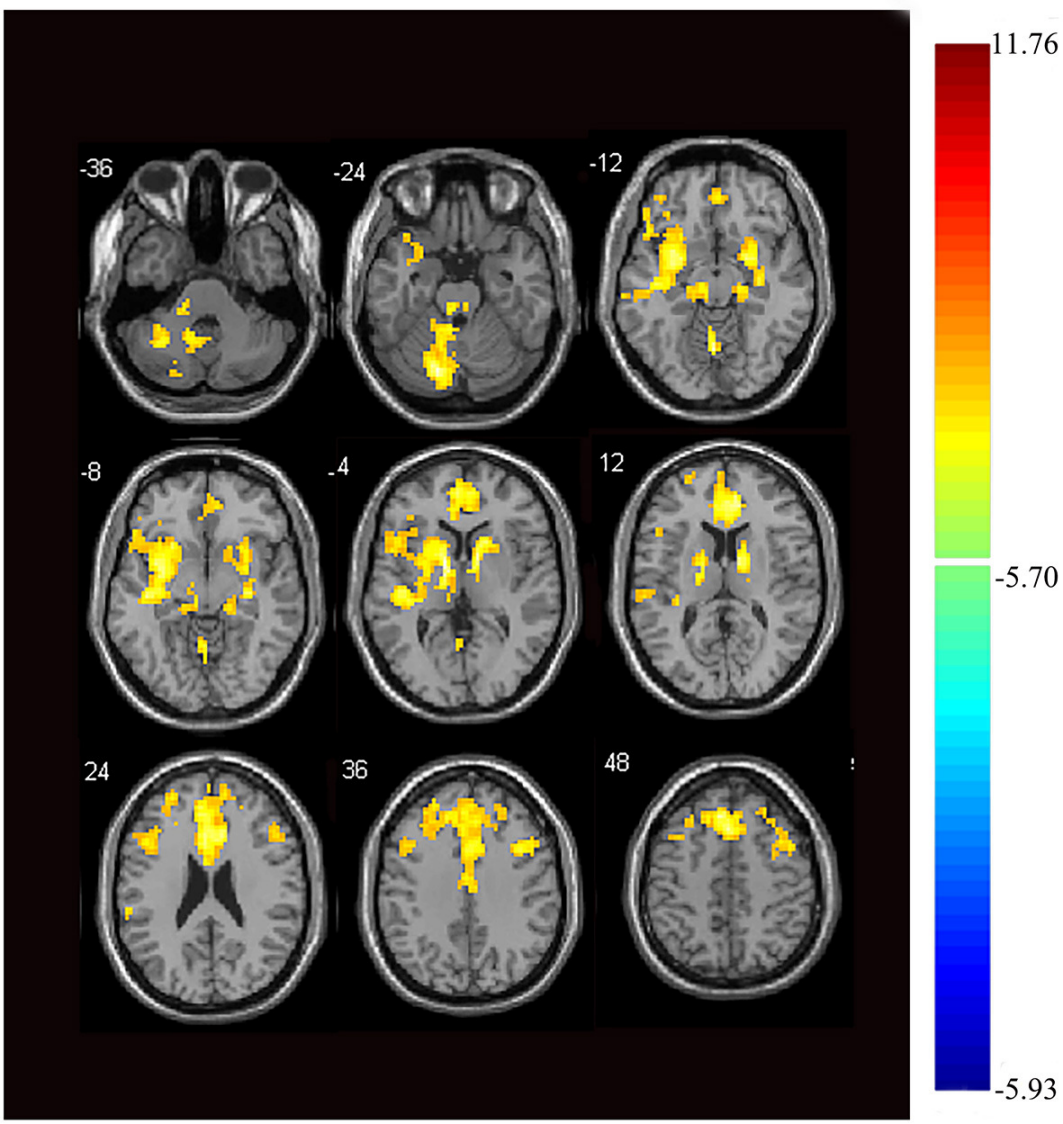
Images showing areas of reduced thalamic functional connectivity in subjects with orofacial pain compared with that of the control group. Subjects with orofacial pain exhibited significantly lower functional connectivity (yellow) in clusters located in the left cerebellum, bilateral anterior cingulate cortices, right parahippocampal gyrus, bilateral middle frontal gyri, right inferior frontal gyrus, bilateral superior frontal gyri, right middle temporal gyrus, right insula, and the left thalamus (*p* < 0.05, family-wise error corrected, voxel > 100). At right, color bars indicate *t*-values.

**Figure 3:**
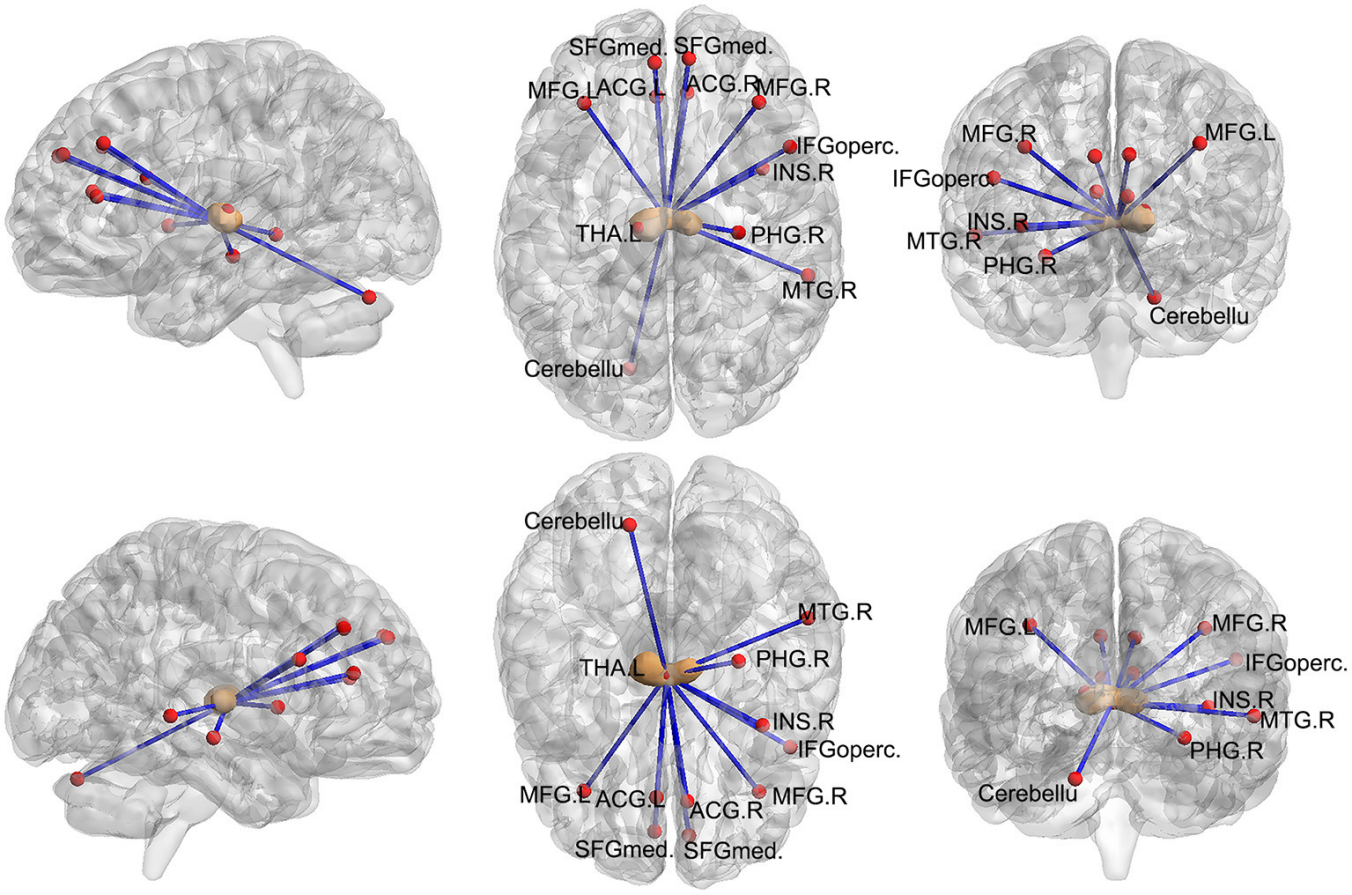
Images showing areas of reduced thalamic functional connectivity in subjects with orofacial pain compared with that in the control group. The brown nodes represent the medial thalamus. The red nodes and blue lines represent decreased FC in participants with orofacial pain relative to control subjects. Subjects with orofacial pain exhibited significantly lower functional connectivity in clusters located in the left cerebellum, bilateral anterior cingulate cortices, right parahippocampal gyrus, bilateral middle frontal gyri, right inferior frontal gyrus, bilateral superior frontal gyri, right middle temporal gyrus, right insula, and the left thalamus (*p* < 0.05, FWE corrected, voxel > 100). ACG, anterior cingulate and paracingulate gyri; PHG, parahippocampal gyrus; MFG, middle frontal gyrus; SFGmed, superior frontal gyrus; MTG, middle temporal gyrus; INS, insula; IFGoperc, inferior frontal gyrus, opercular part; THA, thalamus; FWE, family-wise error.

Next, we examined the FC between the two seeds and the remaining brain regions. Compared with the control group, the seed-based FC analysis of the medial thalamus seed region showed decreased FC to 12 brain regions: the left cerebellum, bilateral anterior cingulate cortex (ACC), right parahippocampal gyrus, bilateral middle frontal gyrus, bilateral superior frontal gyrus, right inferior frontal gyrus, right middle temporal gyrus, right insula, and left thalamus (*p* < 0.05, FWE corrected, voxel > 100; Figures 2, 3). However, we did not find altered FC between the dorsal area of the thalamus and any of the brain regions.

### Correlation Analyses

**Figure 4:**
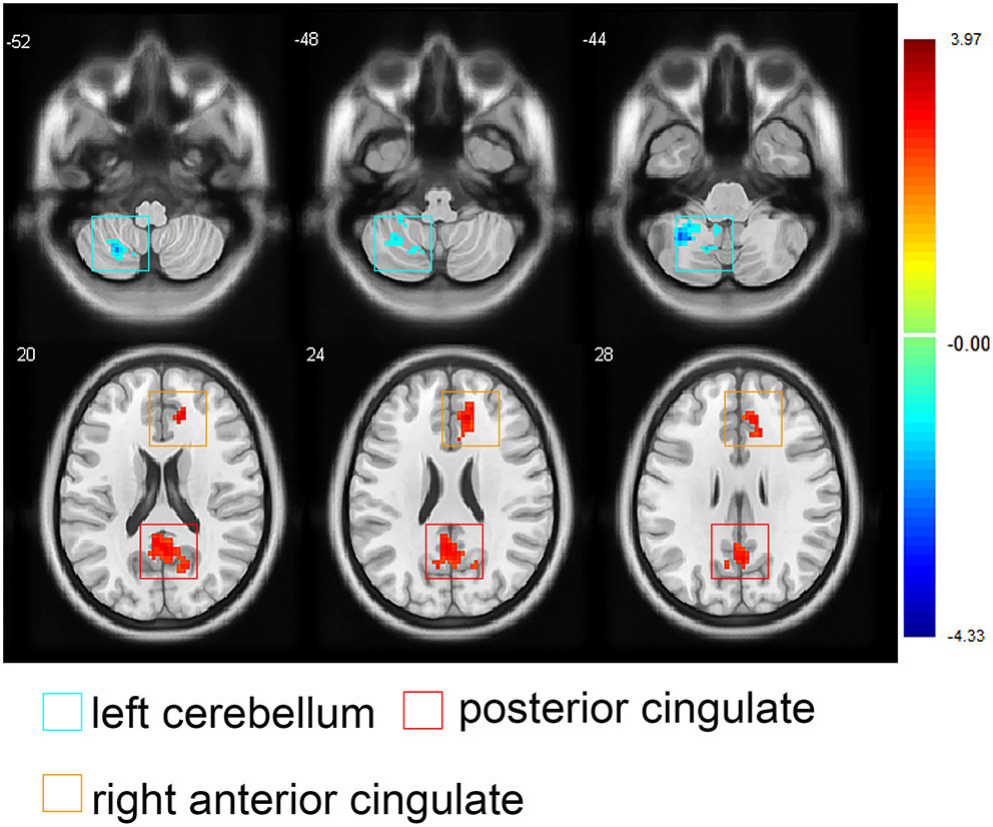
Images showing the correlations between the medial thalamus-seeded functional connectivity and the visual analog scale score changes in participants with orofacial pain. Red areas represent positive correlations (the posterior cingulated cortex and right anterior cingulated cortex), blue areas represent negative correlations (the left superior cerebellum), (AlphaSim correction; note: The threshold for significant clusters was set as follows: 1,000 iterations, *p* < 0.05 at the cluster level combined with *p* < 0.05 at the voxel level, 4 estimated smooth kernels, at least 154 voxels). At right, color bars indicate *t-*values.

In the orofacial pain group, the FC between the medial thalamus, right ACC, and posterior cingulate cortex (PCC) was positively correlated with the VAS score changes, and the FC between the medial thalamus and the left cerebellum was negatively correlated with the VAS score changes (*p* < 0.05, AlphaSim correction, Figure 4 and Table 4).

**Table 4:** Correlations between the medial thalamus-seeded FC and VAS changes in the orofacial pain group.

**Brain region**	**MNI coordinates**			**Clusters size**	***t-*value**
	***x***	***y***	***z***		
**Positive correlation**					
Right ACC	15	15	51	168	4.36
Posterior cingulate cortex	−3	−54	24	337	3.08
**Negative correlation**					
Left cerebellum	−33	−54	−45	166	−4.11

*The FC between the medial thalamus and the right ACC and posterior cingulate cortex (PCC) was positively correlated with the VAS score changes, and the FC between the medial thalamus and the left cerebellum was negatively correlated with the VAS score changes (AlphaSim correction, note: The threshold for significant clusters was set as follows: 1,000 iterations, p < 0.05 at the cluster level combined with p < 0.05 at the voxel level, 4 estimated smooth kernels, at least 154 voxels; Figure 4)*.

## Discussion

In our study, the VAS scores significantly increased after elastic separator placement, indicating that the elastic separators successfully induced orofacial pain to a mild degree (26), and our results showed that thalamic internal function was altered in individuals with orofacial pain. The FC of the medial thalamus significantly differed between the groups. This study provided evidence of the key role of the thalamus in the mechanism of orofacial pain and may shed light on the different roles of the thalamic subregions in orofacial pain perception.

The thalamus is considered to be a critical region in pain transmission and modulation (27). Over the past decade, functional and structural changes were found in the thalamus in patients with orofacial pain (16, 28). Previous studies suggested that the thalamus can be divided into several subregions in different ways (29). For example, the thalamo-cortical pathways can be segregated into lateral and medial pathways, which are mainly involved in sensory discrimination and pain perception, respectively (30). In addition, a previous study provided classification for the thalamic nuclei: sensorimotor group, limbic group, and sensorimotor/limbic bridging nuclei (31). This is similar to our findings: we found different functional activities in different thalamic subregions, that is, decreased fALFF in the medial thalamus and increased fALFF in the dorsal area of the thalamus in participants with orofacial pain. Thus, we speculate that the medial thalamus and dorsal area of the thalamus may play important roles in orofacial pain perception.

The Automated Anatomical Labeling atlas (https://www.oxcns.org/aal.html) was used to determine the anatomical boundaries of the thalamic subregions, and we identified the ventroposterior nucleus located in the dorsal area of the thalamus, which relays the nociceptive information to the cortex. The dorsal area of the thalamus might be associated with orofacial pain sensation, considering the role of the ventroposterior nucleus in sensory-discriminative function (30). Although increased fALFF was observed in the dorsal area of the thalamus, we did not observe any FC changes between the dorsal area of the thalamus and other brain regions in participants with orofacial pain. Further research should be undertaken to investigate its exact role of the dorsal area of the thalamus.

The medial thalamus demonstrated decreased fALFF, and we found a lower FC of the medial thalamus with some regions; interestingly, most of them belong to the prefrontal cortex (PFC) and temporal cortex. The PFC plays a critical role in a range of cognitive processes, including decision-making, working memory, and emotional regulation (32). The temporal cortex is known to be associated with various cognitive functions, such as memory, auditory cognition, and semantics (33). The medial temporal lobe includes the parahippocampal cortex, essential in recognition and source memory (34). Thus, the reduced FC between the thalamus and the PFC may be associated with aberrant emotion regulation and cognition, and the reduced FC between the thalamus and the temporal cortex may be involved in recognition memory in orofacial pain.

Interestingly, the ACC, PFC, insula, temporal cortex, and parahippocampal gyrus are all components of the limbic system. Emotion, memories, and behavior emerge from the coordinated activities of regions connected by the limbic system (35). Moreover, the ACC was reported to receive nociceptive information from the medial thalamus and contribute to the affective and motivational instead of sensory and discriminative aspects of pain (36). In fact, both the ACC and insula have long been considered to be important for encoding the emotional aspects of pain (37). Therefore, the medial thalamus is closely connected to the limbic system and likely plays a vital role in the cognitive and emotional modulation of orofacial pain.

Recently, the cerebellum was reported to both be involved in pain perception and tightly connected with some brain regions involved in cognition (38), and the cerebellum's engagement in pain processing probably modulates the activity of both the somatosensory and cingulate cortices (39). A previous study demonstrated that the cerebellum might be associated with activation of the endogenous pain inhibitory mechanisms (40). In our study, we found negative correlations between the VAS score changes and the medial thalamus-seeded FC in the left cerebellum, indicating that the cerebellum may be associated with pain intensity, but the specific role of the cerebellum in orofacial pain is still an interesting topic worth exploring.

In the present study, the FC between the thalamus and other brain regions involved in acute nociceptive stimuli were not significantly different between the groups, which may be because of the fact that the separator-induced orofacial pain is a type of chronic pain, and the brain activity is confined to emotion-related networks in chronic pain (41).

Positive correlations were found between the VAS score changes and medial thalamus-seeded FC in the right ACC and PCC, suggesting that the orofacial pain intensity is associated with the FC between the medial thalamus and these brain regions, which may be associated with adaptation to orofacial pain. However, there were no significant FC changes between the medial thalamus and the PCC compared with that of the controls. This finding should be further investigated.

This study had some limitations. First, the age range of our participants was narrow (19–23 years); hence, the results are only valid for the youth population and should not be generalized to a broader population. Second, the exploratory correlation analyses of pain intensity and seed-based FC changes were only corrected with the AlphaSim correction in this study; therefore, the correlation we observed should be treated with caution and worth further study as an a priori hypothesis. Third, we only studied orofacial pain induced in healthy subjects, which is relatively simple compared to the actual situation in the clinical population. Future studies must consider studying patients with existing orofacial pain, rather than just healthy subjects with induced orofacial pain, to better explore the central mechanism of orofacial pain. We also found that VAS scores were not zero before the placement of separators, which may either be due to the slight discomfort caused by oral examinations or the subjects' psychological preference for choosing non-zero numbers.

In summary, our study demonstrated alteration in the functional activity in thalamic subregions, suggesting that the medial thalamus and the dorsal area of the thalamus may play important roles in orofacial pain perception, and that the medial thalamus may play an important role in the cognitive and emotional modulation of orofacial pain. The analysis of intrinsic functional changes in orofacial pain by fMRI may be helpful to further understand the mechanism of this disorder and guide the effective treatment.

## Data Availability Statement

The raw data supporting the conclusions of this article will be made available by the authors, without undue reservation.

## Ethics Statement

The studies involving human participants were reviewed and approved by West China Stomatological Hospital of Sichuan University (Sichuan, China). The patients/participants provided their written informed consent to participate in this study.

## Author Contributions

YJ: conceptualization, methodology, data curation, formal analysis, writing—original draft, review, and editing. HY, FZ, and FL: methodology, data curation, formal analysis, writing—original draft, review, and editing. JW and XY: conceptualization, data curation, formal analysis, writing—original draft, review, and editing. HLi: methodology, data curation, writing—original draft, review, and editing. HLo, QG, and WL: conceptualization, methodology, formal analysis, writing—original draft, review, and editing. All authors gave their final approval and agree to be accountable for all aspects of the work.

## Conflict of Interest

The authors declare that the research was conducted in the absence of any commercial or financial relationships that could be construed as a potential conflict of interest.
